# Analysis of satisfaction levels and perceptions of clinical competency: a mixed method study on objective structured clinical examinations in undergraduate dental students

**DOI:** 10.1186/s12909-024-05639-0

**Published:** 2024-06-17

**Authors:** Naseer Ahmed, Samiya Aziz, Rizwan Jouhar, Muneeba Rehmat, Afsheen Maqsood, Resham Nadeem, Laraib Magsi, Artak Heboyan

**Affiliations:** 1 Department of prosthodontics, Altamash Institute of Dental Medicine, Karachi, 75500 Pakistan; 2 Department of Operative Dentistry and Endodontics, Altamash Institute of Dental Medicine, Karachi, 75500 Pakistan; 3 Department of Restorative Dental Science, King Faisal University, Al-Ahsa, 31982 Saudi Arabia; 4 Department of Medical Education, Altamash Institute of Dental Medicine, Karachi, 75500 Pakistan; 5 Department of Oral Pathology, Bahria University Dental College, Karachi, Pakistan; 6 Department of Periodontology, Altamash Institute of Dental Medicine, Karachi, 75500 Pakistan; 7 Department of Research Analytics, Saveetha Institute of Medical and Technical Sciences, Saveetha Dental College and Hospitals, Saveetha University, Chennai, 600 077 India; 8 Department of Prosthodontics, Faculty of Stomatology, Yerevan State Medical University after Mkhitar Heratsi, Str. Koryun 2, Yerevan, 0025 Armenia; 9 Department of Prosthodontics, School of Dentistry, Tehran University of Medical Sciences, North Karegar St, Tehran, Iran

**Keywords:** Objective structured clinical examination, Clinical competencies, Dental students, Student satisfaction, Focus group interviews, OSCE effectiveness

## Abstract

**Objective:**

To analyze the satisfaction levels, perceptions of developing clinical competencies through objective structured clinical examination and to explore the experiences, challenges, and suggestions of undergraduate dental students.

**Methods:**

The study adopted a mixed-method convergent design. Quantitative data were collected from 303 participants through surveys, evaluating satisfaction levels with objective structured clinical examination (OSCE). Additionally, qualitative insights were gathered through student focus group interviews, fundamental themes were developed from diverse expressions on various aspects of OSCE assessments. The Chi-Square tests, was performed to assess associations between variables. Data integration involved comparing and contrasting quantitative and qualitative findings to derive comprehensive conclusions.

**Results:**

The satisfaction rates include 69.4% for the organization of OSCE stations and 57.4% for overall effectiveness. However, a crucial challenge was identified, with only 36.7% of students receiving adequate post-OSCE feedback. Furthermore, a majority of students (50%) expressed concerns about the clinical relevance of OSCEs. The study showed a significant associations (*p* < 0.05) between satisfaction levels and years of study as well as previous OSCE experience. Student focus group interviews revealed diverse perspectives on OSCE assessments. While students appreciate the helpfulness of OSCEs, concerns were raised regarding time constraints, stress, examiner training, and the perceived lack of clinical relevance.

**Conclusion:**

The students anticipated concerns about the clinical relevance of OSCEs, highlighting the need for a more aligned assessment approach. Diverse perspectives on OSCE assessments reveal perceived helpfulness alongside challenges such as lack of feedback, examiner training, time constraints, and mental stress.

**Supplementary Information:**

The online version contains supplementary material available at 10.1186/s12909-024-05639-0.

## Introduction

Objective Structured Clinical Examination (OSCE) have gained significant recognition as an assessment method in dental education [1]. The assessment method was initially developed in the medical field and has since been adapted and implemented in dental education due to its effectiveness in evaluating clinical skills and competencies [2]. Traditionally, dental education heavily relied on conventional exams such as short essay questions, short answer questions, true and false assessments, and practical demonstrations to evaluate students’ clinical proficiency. However, these methods often lacked standardization, limited feedback, inability to assess real-world skills, time-consuming, narrow focus or rote memorization, and objective evaluation, leading to potential discrepancies in assessment outcomes. OSCEs emerged as a solution to address these challenges by providing a structured and standardized approach to clinical assessments [3].

The implementation of OSCEs in dental education offers several potential benefits. Firstly, it provides a standardized assessment format that ensures fairness and consistency across students [4]. This format reduces bias and subjectivity in evaluations, promoting more reliable and valid assessments outcome. Additionally, OSCEs help students develop time management skills and adaptability, simulating challenges encountered in real clinical settings [5]. The structured nature of OSCEs encourages students to develop these skills by providing a controlled environment that mirrors real-world scenarios [6]. Moreover, OSCEs allow students to receive immediate feedback on their performance, facilitating self-reflection, and identifying areas for improvement [7]. However, it is important to mention assessment of values (affective domain) within OSCEs. This examination goes beyond mere technical skills and encompasses elements such as professionalism, ethical decision making, and patient-centered care. Evaluating these domains adds another layer of complexity to the assessment process, aiming to cultivate well-rounded dental professionals capable of delivering high-quality care [8]. Despite the potential advantages, it is essential to explore the effectiveness of OSCEs in dental education, particularly in terms of student satisfaction and the development of clinical competencies. Understanding students perceptions and experiences regarding OSCEs can provide valuable insights into the strengths and weaknesses of this assessment method. Additionally, investigating the impact of OSCEs on the development of clinical competencies can inform educational strategies and contribute to the continuous improvement of dental curriculum.

Objective Structured Clinical Examinations have been widely investigated and implemented in various healthcare disciplines, including dental education. Hodges [9] 2009 conducted a study on OSCEs in clinical education and found that OSCEs demonstrated higher reliability and validity for assessing clinical competencies, including those specific to dental education. Similarly, Schoonheim-Klein et al. [10] compared the performance of dental students in OSCEs and traditional clinical exams. They reported that OSCEs provided a more standardized and comprehensive assessment of clinical skills, highlighting their effectiveness in evaluating competencies such as history taking, communication, and treatment planning. Myyry et al. [11] explored the impact of OSCEs on dental students learning experiences. They found that OSCEs promoted active learning, self-reflection, and improved confidence in clinical skills. Students perceived OSCEs as valuable assessments that enhanced their clinical competence.

Clinical competence encompasses not only skills and abilities but also values and knowledge essential for effective patient care [8]. Thus, OSCE should aim to comprehensively assess these diverse domains. In addition to evaluating procedural proficiency, an OSCE should incorporate scenarios that challenge students to demonstrate their ethical decision-making, communication skills, and understanding of fundamental principles in patient care [11]. By encompassing a holistic approach to clinical competence assessment, OSCEs can better prepare students for the complex realities of dental practice. This integration of values, knowledge, and skills within the OSCE framework is crucial for ensuring that graduates are not only technically proficient but also compassionate and ethically grounded dental care professionals [4, 10].

Park et al. [12] explored the challenges faced by dental students in OSCEs. They identified time pressure, anxiety, and difficulty in demonstrating clinical skills within the limited station time as significant challenges. Providing adequate preparation and guidance to students was highlighted as crucial for optimal performance in OSCEs. Chimea et al. [13] and Sadia et al. [14] discussed the limitations of OSCEs in healthcare. The limitations included the cost and resources required for implementation, the potential for examiner variability, and the limited ability to assess complex cognitive skills or clinical judgment.

The current understanding of the experiences and perceptions of undergraduate dental students regarding (OSCEs) in dental education is primarily based on quantitative assessments, however, the available data is somewhat limited, urging the necessity for a mixed-method study. By integrating quantitative measures of student performance and satisfaction levels with qualitative insights into their experiences, challenges, and suggestions, a more comprehensive understanding of the effectiveness and implementation of OSCE in undergraduate dental education can be achieved.

The working hypothesis of the quantitative part of this study was whether there is a significant association between students’ perceived clinical competence and their satisfaction with OSCEs, considering their year of study and previous OSCE experience. In this mixed-method study, the qualitative part typically does not have specific hypotheses like the quantitative part. Instead, the qualitative component intended to explore, understand, and generate in-depth insights and interpretations regarding the satisfaction and clinical competency level of students.

The study outcome will contribute to the existing literature on the effectiveness of OSCE in dental education, providing comprehensions into student satisfaction and the development of clinical competencies. The findings can inform educators and policymakers in refining and optimizing OSCE assessment practices in dentistry, enhancing the educational experience for dental students. This study aimed to analyze the satisfaction levels and perceptions of developing clinical competencies of OSCE and to explore the experiences, challenges, and suggestions of dental students regarding OSCEs as an assessment method in undergraduate dental students.

## Materials and methods

### Study design and ethical consideration

This study adopted a mixed-methods convergent design, incorporating both quantitative and qualitative data collection and analysis methods. The methodological orientation of qualitative part of the study was based on an empirical phenomenological approach. The study was conducted from April to October 2023. The study adopted COREQ (consolidated criteria for reporting qualitative research) criteria for reporting, (Supplementary file 1). Ethical approval was obtained from the ethical review committee of the Altamash institute of dental medicine, Karachi, Pakistan before data collection (AIDM/ERC/07/2023/02). A written Informed consent incorporated in the questionnaire was obtained from all participants, ensuring their voluntary participation and the confidentiality of their responses.

### Sample size estimation

The sample size was calculated using Open-Epi software version 3.01. Considering a population size (N) of 1160, a hypothesized % frequency of the outcome factor in the population (p) at 50% +/- 5%, a confidence level of 95%, and a margin of error of 5%, the estimated sample size was determined to be 303 for the quantitative part of the study. Additionally, for the qualitative component, 45 participants were selected for focused group interviews based on data saturation and sampling continuity.

### Participant selection and subject criteria

A purposive sampling technique was used to select dental students who have completed OSCE assessments.

**Inclusion Criteria**:

- Undergraduate dental students enrolled in a dental program.
- Students who have completed OSCE assessments.
- Students who are willing to participate voluntarily and provide informed consent.
- Students from different academic years or stages of the dental program.

**Exclusion Criteria**:

- Graduate or postgraduate dental students.
- Dental students who have not yet been exposed to OSCE assessments.
- Students who have already participated in a similar study on OSCEs in dental education.

### Data collection

The well-constructed questionnaire was distributed online through google® platform. The target population was the students of Bachelor of Dental Surgery (BDS) studying at Altamash Institute of dental medicine, Karachi, Pakistan. The students were contacted through WhatsApp® and emails for voluntary participation in the study. For the focused group sessions, a total of forty five 3rd and 4th year BDS students were divided into 5 focused group interview sessions on Zoom® platform. To minimize selection bias, participants were assigned to groups using a “random assignment approach”. Each participant were given an equal chance of being assigned to any of the groups.

#### Quantitative data

A questionnaire was developed (supplementary file 2) focusing on student satisfaction with OSCEs and perceived impact on clinical competencies. The form utilized Likert [15] scale to gather quantitative data. The participants were asked to rate their level of agreement or perception on a 5-point Likert scale, ranging from “Strongly Disagree” to “Strongly Agree” for assessing satisfaction with various aspects of the OSCE assessment method. Similarly, the Likert scale was also used to gauge the extent to which OSCEs contributed to the development of clinical competencies, ranging from “Not at all” to “Extremely.” The Likert scale was used to provide students with a range of options to express their opinions, allowing for a more detailed understanding of their perspectives.

#### Qualitative data

Focus group interview was conducted according to the list of prompts (supplementary file 3) in a subset of students to obtain qualitative data on their experiences and perceptions of OSCE. Five focus group interviews was conducted altogether with 9 participants in each group from the dental institute. Moderators S.A, R.N, and M.R (BDS degrees and designation of clinical demonstrators holding an experience of over 6 years) who were not a part of the OSCE assessments (as an invigilator and examiner) in the institute to avoid biasness conducted the interviews. Audio and video recordings and detailed notes were taken during the discussions. Permission was obtained from the school to hold the interviews after the college time. Each interview lasted between 30 and 40 min. The repeat interviews were not carried out and point of saturation during the online sessions on specific prompts were given consideration. The research team did transcription manually. Manual transcription involved listening to the recordings and typing out the spoken words precisely, including any pauses, and tones of speech, (Supplementary file 2). To ensure the credibility and trustworthiness of the qualitative findings, member checking was employed. Following each focus group interview, the research team (N.A; S.A) shared a summary of the key themes and interpretations with the participants via email. Participants were invited to review the summary and provide feedback on its accuracy and completeness. This member-checking process allowed participants to verify that their experiences were accurately represented and strengthened the credibility of the qualitative data.

Prior to the interviews, the team Moderators (M) met with each group of students and briefed them on the nature of the interview and objective of the study, explaining that they will be free to talk and ask questions if they did not understand some of the issues during discussion. The focus group interviews conducted in our study were adopted from Green and Hart (1999) [16] and Ho Debbie [17], in terms of group set-up and analysis procedure.

### Validity and reliability of the research tool

#### Validity of the Questionnaire

The research team, along with an expert member from the medical education and clinical faculty, assessed the questionnaire to ensure face and content validity.

#### Reliability of the questionnaire

The reliability of the items was performed through Cronbach’s alpha for internal consistency (α = 0.81). A pilot study was conducted using a subset of the total sample size, typically around 20% of the students. The purpose of the pilot study was to test the questionnaire on a smaller scale to identify any potential issues with item clarity, wording, or response format. The data collected from the pilot study was analyzed to assess the students’ responses and the reliability of the questionnaire. Any necessary modifications or adjustments required were addressed before administering the questionnaire to the entire sample.

### Data analysis

Descriptive statistics was used to analyze the survey responses, including frequencies, percentages, and mean scores. The data was subjected to normality testing through Shiparowilk test. Inferential statistical tests Chi- square, was employed to explore relationships between student satisfaction levels and clinical competency development with year of study and previous OSCE experience. A *p*-value of ≤ 0.05 was deemed as significant.

Inductive thematic analysis was conducted on the transcriptions of the focus group discussions. The research team, along with a research assistant familiar with qualitative methods, coded the data independently for initial themes. Discrepancies in coding were resolved through discussion to ensure intercoder reliability. The identified themes were then reviewed by the entire research team to enhance the credibility of the analysis.

## Results

### Quantitative analysis

The mean age of participants was 21.42 ± 1.66. The gender distribution consisted of 84 (27.7%) male and 219 (71.9%) female studying in the local dental institute. In terms of academic progression, participants were spread across the 3rd and 4th year BDS having the representation at 57.75% (175 participants). The distribution of 1st and 2nd year BDS was 42.24% (128) students in the quantitative component of study. Table 1.

**Table 1:** Demographic details of the participants (*n* = 303)

Variable		*n*%
Gender	Male	84 (27.7%)
	Female	219 (71.9%)
	1st and 2nd BDS	128 (42.24%)
	3rd and 4th BDS	175 (57.75%)

BDS: bachelor of dental surgery, OSCE: objective structured clinical examinations

Table 2 describes student satisfaction with the OSCE. Particularly, students across different academic years and with varying OSCE exposure show distinctive patterns of satisfaction. Chi-Square analysis indicated a significant association (*p* < 0.05) between various aspects of OSCE satisfaction. For instance, regarding clarity of instructions, 65.3% of participants either agreed or strongly agreed, and there was a statistically significant association with both year of study (*p* = 0.001) and previous OSCE experience (*p* = 0.020). Similarly, for the organization and flow of OSCE stations, 69.4% agreed or strongly agreed, with a significant association with both year of study (*p* = 0.001) and previous OSCE experience (*p* = 0.001). The fairness of the assessment process showed a satisfaction rate of 65.4%, and there was a statistically significant association with year of study (*p* = 0.001). In terms of the adequacy of time provided, 58.7% agreed or strongly agreed, and there was a significant association with year of study (*p* = 0.001). The relevance of OSCE stations to clinical practice saw a satisfaction rate of 50%, with a significant association with year of study (*p* = 0.001). Feedback after the OSCE received positive responses from 36.7%, and there was a significant association with both year of study (*p* = 0.001) and previous OSCE experience (*p* = 0.001). Regarding the overall effectiveness of OSCEs in assessing clinical competencies, 57.4% agreed or strongly agreed, with a significant association with year of study (*p* = 0.001). Nevertheless, no significant difference was observed in students satisfaction levels when the responses relevance of OSCE stations to clinical practice (*p* = 0.186), fairness of the assessment process (*p* = 0.717), adequacy of time provided for each OSCE station (*p* = 0.223), and overall effectiveness of OSCEs in assessing clinical competencies (*p* = 0.304) were analyzed in students with a prior OSCE experience. However, overall the students indicate generally positive responses with variations in satisfaction levels across different aspects of the OSCE.

**Table 2:** Relationship of student satisfaction with year of study and previous OSCE experience (*n* = 303)

Variable	SDA (*n*%)	DA (*n*%)	*N* (*n*%)	A (*n*%)	SA (*n*%)	Year of study (*p*-value)		Previous experience with OSCE (*p*-value)
Clarity of instructions provided before each OSCE station	2 (0.8)	11 (3.6)	92 (30.4)	126 (41.5)	72 (23.8)	0.001		0.020*
The organization and flow of the OSCE stations	5 (1.7)	10 (3.3)	78 (25.7)	146 (48.2)	64 (21.2)	0.001		0.001*
The fairness of the assessment process	6 (2.0)	9 (3.0)	90 (29.7)	119 (39.3)	79 (26.1)	0.001		0.717
The adequacy of time provided for each OSCE station	20 (6.6)	39 (12.9)	66 (21.8)	97 (32.0)	81 (26.7)	0.001		0.223
The relevance of the OSCE stations to the clinical practice	8 (2.6)	23 (7.6)	121 (39.9)	85 (28.1)	66 (21.8)	0.001		0.186
The helpfulness of feedback received after the OSCE	19 (6.3)	47 (15.5)	126 (41.6)	65 (21.5)	46 (15.2)	0.001		0.001*
The overall effectiveness of OSCE in assessing clinical competencies	3 (1.0)	30 (9.9)	96 (31.7)	96 (31.7)	78 (25.7)		0.001	0.304

1 = Strongly Disagree (SDA), 2 = Disagree (DA), 3 = Neutral (N), 4 = Agree (A), and 5 = Strongly Agree (SA), *p-value ≤ 0.05 was considered significant

Table 3 demonstrates the distribution of perceptions regarding the development of clinical competencies among participants. The distribution of responses shows noteworthy trends across different competency domains. There was a substantial consensus on the enhancement of diagnostic skills, with 71.5% expressing moderate to extreme development. Treatment planning abilities also lay down positive responses, with 61.1% indicating moderate to extreme enhancement. Technical proficiency in dental procedures was evident by 81.1% reporting moderate to extreme development, and statistically significant (*p* = 0.001) concerning years of study. Positive feedback regarding communication skills with patients was noted from 70.2% of participants, with a statistically significant association with years of study (*p* = 0.001). Acknowledgment of the development of professionalism and ethical conduct was expressed by 69.1% of participants. Critical thinking and problem-solving skills were recognized by 55.0%, and a statistically significant association was found with years of study (*p* = 0.001) and previous OSCE experience (*p* = 0.002). Regarding time management during patient care, 65.2% reported moderate to extreme development, and teamwork with other healthcare professionals received positive feedback from 71.2%. Both these responses showed statistically significant relationship with years of study (*p* = 0.001).

**Table 3:** Relationship of perceptions of clinical competency development with years of study and previous experience with OSCE (*n* = 303)

Variable	NAA (*n*%)	S (*n*%)	M (*n*%)	VM (*n*%)	E (*n*%)	Year of study (*p*-value)	Previous experience with OSCE (*p*-value)
Diagnostic skills	9 (3.0)	29 (9.6)	103 (34.0)	113 (37.3)	49 (16.2)	0.001	0.158
Treatment planning abilities	9 (3.0)	21 (6.9)	145 (47.9)	88 (29.0)	40 (13.2)	0.001	*0.042
Technical proficiency in performing dental procedures	20 (6.6)	19 (6.3)	138 (45.5)	108 (35.6)	18 (5.9)	0.001	*0.001
Communication skills with patients	9 (3.0)	57 (18.8)	88 (29.0)	125 (41.3)	24 (7.9)	0.001	*0.014
Professionalism and ethical conduct	5 (1.7)	46 (15.2)	82 (27.1)	125 (41.3)	45 (14.9)	0.001	0.073
Critical thinking and problem solving skills	2 (0.8)	21(6.9)	111 (36.6)	117 (38.6)	54 (17.8)	0.001	*0.002
Time management during patient care	20 (6.6)	22 (7.3)	65 (21.5)	131 (43.2)	65 (21.5)	0.001	*0.001
Teamwork and collaboration with other healthcare professionals	26 (8.6)	55 (18.2)	101 (33.3)	90 (29.7)	31 (10.2)	0.001	0.076

NAA: Not at all, S: Slightly, M: Moderately, VM: Very much, E: Extremely, *statistically significant associations are indicated by *p*-values < 0.05

### Qualitative analysis

Table 4 outlines nine fundamental themes derived from student focus group discussions on OSCE assessments. The qualitative analysis in this study employed an inductive thematic analysis approach. The identified themes include; efficiency in preparation and scoring, challenges in knowledge and clinical proficiency evaluation, considerations about assessment content and practicality, integration with the curriculum, feedback and evaluation experiences, comparisons with other exam formats, perspectives on OSCE contribution to future clinical practice, and insights on continuous improvement in evaluation and logistics. These themes were derived from predefined prompts (P) subthemes and associated direct quotes from student interviews. The prompts (P) served as discussion topics, and the corresponding responses, quantified in terms of frequency based on the 45 participants. Prompt 1 direct quotes indicates that 14 (31.11%) of students find OSCE helpful but time-consuming in terms of preparation, while 12 (26.67%) express stress and nervousness, particularly in the context of facing 4 to 16 OSCE exams. P2 highlights that 24 (53.33%) of students perceive OSCE as easy to pass, and 21 (46.67%) believe they enhance knowledge and relevant clinical skills. P3 reveals concerns, with 15 (33.33%) expressing reservations about time limitations and 20 (44.44%) feeling that OSCE mostly assess knowledge. P4 reflects diverse views, with 16 (35.56%) of students seeing it as somehow assessing clinical skills effectively, and 15 (33.33%) noting their relation to real-life scenarios. P5 shows that 18 (40%) of students believe OSCE are integrated with the current curriculum, while 17 (37.78%) feel the curriculum needs an update. The P6 captures opinions on feedback provision, with 13 (28.89%) stating that no feedback was provided post-exam, and 16 (35.56%) advocating for feedback. Prompt 7 explores comparisons, with 19 (42.22%) finding OSCEs better than other forms of exams, and 07 (15.56%) expressing that OSCEs accurately represent clinical skills. Prompt 8 and P9 discussed various aspects, including 08 (17.78%) believing OSCEs are good for future dental practice and 05 (11.11%) suggesting that OSCEs should be more clinically based and 06 (13.33%) believed that it is important to train the examiner for a standard OSCE.

**Table 4:** Distribution of themes, subthemes, and direct quotes responses from student focus group interviews

*P*	Themes	Subthemes	Direct quotes	Frequency of student expressions (*N* = 45)
P1	Student experience and perceived challenges	OSCE preparation	OSCEs are helpful but lengthy, I spent a lot of time preparing for them	14 (31.11%)
		Examination stress and anxiety	I find OSCEs to be stressful and nerve-racking, especially when facing multiple exams	12 (26.67%)
		Varied experiences	I have had varied experiences across 4 to 16 OSCE exams, each presenting its own set of challenges and learnings	19 (42.22%)
P2	Efficiency in preparation and scoring	Efficiency and scoring	OSCEs are easy to pass and score	24 (53.33%)
		Knowledge and skill development	I feel they have enhanced my knowledge and clinical skills	21 (46.67%)
P3	Knowledge and clinical proficiency evaluation	Time concerns	There is a need for more time during OSCEs, and they mostly assess theoretical knowledge	15 (33.33%)
		Fair time allocation in skill based stations	The time should be allocated based on station content to ensure fairness and accuracy in assessment	20 (44.44%)
		Clinical skills focus	OSCEs are focused on the assessment of clinical skills, ensuring a comprehensive evaluation of students’ practical abilities	10 (22.22%)
P4	Assessment content and practicality	Potential for clinical skills assessment	I believe that the ongoing OSCEs are capable of somehow assessing our clinical skills effectively	16 (35.56%)
		Varied station relevance	I witnessed that some OSCE stations were related well to the real-life scenarios, while others seem disconnected	07 (15.56%)
		Realistic scenarios enhances assessment	I strongly feel that many OSCE stations were very much based on real-life scenarios, which enhances the authenticity of the assessment	15 (33.33%)
		Artificial scenarios reduce validity	I find that OSCE stations were not related to real-life scenarios, which can make us away from the clinical practice we will encounter	07 (15.56%)
P5	Integration with curriculum	Curriculum integration benefits	OSCE were integrated with our current curriculum, which is beneficial for our learning	18 (40%)
		Curriculum disconnection	Unfortunately, OSCE were not integrated with our curriculum, which creates a disconnect between what we learn and what was assessed	10 (22.22%)
		Curriculum alignment needs	The curriculum needs an update to better align with the OSCE format and requirements	17 (37.78%)
P6	Feedback and evaluation	Lack of feedback	No feedback was provided post-exam, which makes it challenging to understand our areas for improvement	13 (28.89%)
		Importance of feedback	I believe that feedback should definitely be provided after the OSCE	16 (35.56%)
		Perceptions of fairness	I felt the OSCE evaluation was fair	07 (15.56%)
			I have mixed feelings about the fairness of the OSCE evaluation, I would say it was partially fair	03 (6.67%)
			I am concerned about the consistency of OSCE evaluation, which was lacking.	06 (13.33%)
P7	Comparison with other exam formats	Ease and effectiveness of OSCE	I prefer OSCEs, practical tests over theory paper stress	19 (42.22%)
		Knowledge assessment in OSCE	While OSCEs test practical skills, they still rely heavily on memorizing facts. I prepared for weeks just for this one exam	09 (20%)
		Accuracy of OSCE in reflecting clinical skills	OSCE stations were like real-life scenarios. They truly showed how well I can perform under pressure in a clinical setting	07 (15.56%)
		Limitations of OSCE in reflecting clinical skills	OSCEs were too artificial. They do not capture the complexities of real patient interactions and unpredictable situations we face as dental professionals	10 (22.22%)
P8	Preparation for future clinical practice	Future practice	Limited value for the complexities of future dental practice	08 (17.78%)
			OSCEs were not good for future dental practice	06 (13.33%)
		Knowledge enhancer	I found OSCEs prepared us for knowledge of dental diseases	09 (20%)
		Motor skills	It is somehow building a foundation for my dental motor skills	04 (8.89%)
		Alternative assessment arrangements	I believe that simulators are a better way to practice dental procedures	08 (17.78%)
		Communication skills	It is falling short on communication skills training	10 (22.22%)
P9	Continuous improvement in evaluation and logistics	OSCE improvements	I believe it needs continuous evaluation and update	08 (17.78%)
			It is a better assessment method and better prepare us for future clinics	10 (22.22%)
			There should be more clinical focus in OSCEs to bridge the theory-practice gap	05 (11.11%)
		OSCE timing and administration	OSCE should be align with theory exams for optimal timing	04 (8.89%)
			It would be fair if time allotment is planned according to station complexity	07 (15.56%)
		Examiner training and standardization	We should provide examiner training to ensure OSCE consistency	06 (13.33%)
			OSCE materials should be standardize, a similar chance should be provided for all examinees	05 (11.11%)

P: prompt, N: number of participants, OSCE: objective structured clinical examinations

### Integration of quantitative and qualitative data through comparison

The majority of students agreed or strongly agreed that the instructions were clear (66%), the OSCE stations were well-organized and ran smoothly (69.4%), the assessment process was fair (65.4%), and the time provided was adequate (58.7%). Although, only half of the students agreed or strongly agreed that the OSCE stations were relevant to clinical practice (50%), and only 36.7% of students agreed or strongly agreed that they received enough feedback after the OSCE. Additionally, students had mixed responses on whether the OSCEs helped them to develop their critical thinking and problem-solving skills (55.0%). The qualitative data revealed that the students generally felt that the OSCEs were an effective way to assess clinical competencies (57.4%). They also reported that the OSCEs helped them to develop their diagnostic skills (71.5%), treatment planning abilities (61.1%), technical proficiency in dental procedures (81.1%), communication skills with patients (70.2%), professionalism and ethical conduct (69.1%), time management skills (65.2%), and teamwork skills (71.2%). However, some students expressed concerns about the time pressure and the relevance of the OSCE stations to clinical practice Table 5.

**Table 5:** Integration of quantitative and qualitative data

OSCE Satisfaction	Quantitative Data	Qualitative Data
Clarity of instructions	66% of students agreed or strongly agreed	Students found the instructions to be clear, but some expressed concern about the time pressure.
Organization and flow of OSCE stations	69.4% of students agreed or strongly agreed	Students found the OSCE stations to be well organized and flowing smoothly.
Fairness of assessment process	65.4% of students agreed or strongly agreed	Students generally felt that the OSCE assessment process was fair.
Adequacy of time provided	58.7% of students agreed or strongly agreed	Some students felt that there was not enough time to complete all of the OSCE stations.
Relevance of OSCE stations to clinical practice	50% of students agreed or strongly agreed	Students had mixed opinions on the relevance of the OSCE stations to clinical practice.
Feedback after the OSCE	36.7% of students agreed or strongly agreed	Students would like to receive more feedback after the OSCE.
Overall effectiveness of OSCEs in assessing clinical competencies	57.4% of students agreed or strongly agreed	Students generally felt that OSCEs were an effective way to assess clinical competencies.
**Clinical Competency**	**Quantitative Data**	**Qualitative Data**
Diagnostic skills	71.5% of students reported moderate to extreme development	Students felt that the OSCEs helped them to develop their diagnostic skills.
Treatment planning abilities	61.1% of students reported moderate to extreme development	Students felt that the OSCEs helped them to develop their treatment planning abilities.
Technical proficiency in dental procedures	81.1% of students reported moderate to extreme development	Students felt that the OSCEs helped them to develop their technical proficiency in dental procedures.
Communication skills with patients	70.2% of students reported moderate to extreme development	Students felt that the OSCEs helped them to develop their communication skills with patients.
Professionalism and ethical conduct	69.1% of students reported moderate to extreme development	Students felt that the OSCEs helped them to develop their professionalism and ethical conduct.
Critical thinking and problem-solving skills	55.0% of students reported moderate to extreme development	Students had mixed opinions on whether the OSCEs helped them to develop their critical thinking and problem-solving skills.
Time management during patient care	65.2% of students reported moderate to extreme development	Students felt that the OSCEs helped them to develop their time management skills.
Teamwork with other healthcare professionals	71.2% of students reported moderate to extreme development	Students felt that the OSCEs helped them to develop their teamwork skills.

## Discussion

The domain of dental education is in a constant state of change, and we must adapt the assessments methods we use to analyze our students clinical competence accordingly. The OSCEs emerge as a noteworthy option, providing a well-organized and standardized method for assessing students’ preparedness to face the dynamic challenges of clinical practice. In this study on students’ satisfaction with objective structured clinical examinations and their efficacy in assessing clinical competencies among dental students, our findings reveal a predominantly positive response on the assessment tool. More than 65% of students expresses agreement on various aspects, such as clarity of instructions, organizational flow, fairness, relevance to clinical practice, and overall effectiveness. Furthermore, the study found that students generally feel that the OSCEs helped them to develop their clinical competencies. 55% of students reported moderate to extreme development in all competency domains, with the most significant development observed in perceived technical proficiency. These findings support the purpose of the study, which was to assess the effectiveness of OSCEs in promoting the development of clinical competencies among dental students. The study hypothesis proved that there is a significant association between OSCE as a tool for evaluating perceived clinical competencies and its satisfaction with student’s level and prior experience.

An examination of the study findings within the context of existing literature reveals noteworthy consistencies and affirms the positive perceptions of Objective Structured Clinical Examinations (OSCEs) among dental students. This aligns with prior studies conducted by Alkhateeb et al. [18] and Egloff-Juras et al. [19] which reported similar findings toward OSCEs among students. The similarity in these findings emphasizes the widespread acceptance and appreciation of OSCEs as an assessment method in dental education. Furthermore, this study contributes to the existing body of knowledge by reinforcing the well-documented effectiveness of OSCEs in evaluating clinical competencies. This is in line with research by Azer SA [20] and Chen et al. [21], both highlighted the utility of OSCEs in comprehensively assessing clinical skills. The observed positive trends in diagnostic skills, treatment planning abilities, and technical proficiency reported in our study highlighted that; OSCEs effectively contribute to the development of practical competencies among dental students. These findings collectively strengthen the argument for the continued use and refinement of OSCEs as a valid and reliable tool for assessing clinical competencies in dental education.

Our study findings highlighted on the multifaceted aspects of student perceptions regarding OSCEs in dental education. The reported high satisfaction levels among participants, especially regarding clarity of instructions, organization of OSCE stations, and fairness of the assessment process, align with the positive outcomes observed in similar studies (Moult A [22] and Rawlings MA [23]). However, it is essential to note the reported satisfaction rate of 36.7% for feedback after the OSCE, indicating a potential area for improvement. This finding is in line with the published literature, where feedback provision in OSCEs has been identified as a common challenge, affecting students’ learning and self-assessment (Wardman MJ [24] and Rees et al. [25]). Thus, the study emphasizes the importance of refining feedback mechanisms in OSCEs to enhance their effectiveness as a formative assessment tool.

The perceived development of clinical competencies among participants, particularly in diagnostic skills, treatment planning abilities, and technical proficiency, reflects positively on the educational impact of OSCEs. Comparable studies (Chen et al. [21] and Zhang et al. [26]) have reported similar trends, emphasizing the utility of OSCEs in developing practical skills and knowledge application. However, the study’s identification of concerns related to time limitations and the predominant assessment of knowledge in OSCEs is in line with the broader discourse on the challenges associated with this assessment method (Boursicot et al. [27] and Brannick et al. [28]). This highlights the need for a balanced approach in OSCE design, ensuring that both cognitive and practical aspects are adequately addressed to provide a comprehensive evaluation of students’ clinical competencies.

This study despite of several strengths met some limitations. Firstly, the study was conducted at a single dental school, the sample may not adequately represent the diversity of students, faculty, resources, or teaching methods found across different educational institutes. Secondly, the study relied on self-reported data from students, which may be subject to bias. Thirdly, the study did not directly measure clinical competencies, but rather assessed students’ perceptions of their development. Lastly, the initial two years of BDS studies do not exclusively conduct real OSCE exams; rather, emphasis is given to practical skills assessment. Therefore, students may have limited knowledge, exposure, and perception compared to clinical years. Furthermore, OSCE as an assessment tool was not compared with other forms of assessment. Future research should address these limitations by conducting multi-institutional studies, using various assessment tools, and objective measures of clinical competencies.

The choice to conduct focus groups on the Zoom® platform represents a pragmatic response to logistical challenges and the need for remote engagement. While this virtual format provides accessibility and inclusivity, the literature on virtual qualitative research suggests potential alterations in group dynamics and data richness. The absence of non-verbal cues and the influence of technological issues may impact participant interaction^,^ effecting group dynamics, and the reliance on self-reported data, which may introduce bias [29]. Regardless of these challenges, our study leveraged the advantages of virtual platforms, ensuring efficient data collection. The findings should be interpreted in consideration of the methodological implications introduced by the virtual setting, prompting future research to explore strategies for optimizing virtual qualitative data collection.

Despite the shortcomings, this study provides valuable insights into student satisfaction with OSCEs and their effectiveness in assessing clinical competencies. The findings suggest that it is a valuable tool for assessing clinical competencies in dental students and that OSCE can promote the production of well-rounded dental professionals. The qualitative analysis in this study demonstrated strengths in adhering to the COREQ [30] criteria, ensuring transparency, and promoting participant engagement. The rigorous application of qualitative research standards facilitated a comprehensive exploration of students’ perceptions. To enhance the effectiveness of OSCEs, it is recommended to provide detailed and personalized feedback to students post-OSCE, to promote a conducive learning environment. The relevance of OSCE stations to clinical practice can be improved by incorporating real-life scenarios and contemporary medical challenges. Addressing time pressure concerns during the exam is crucial to reduce stress and allow an authentic skill demonstration. Exploring alternative methods for assessing critical thinking and problem-solving skills, such as case-based assessments or simulation exercises, could provide valuable conception. Further research to identify factors contributing to student satisfaction with OSCEs through surveys and interviews is essential for continual improvement. Future studies could integrate these recommendations, focusing on their combined impact, and longitudinally assess the sustained benefits on students’ clinical performance, contributing to the ongoing optimization of OSCE in medical education.

## Conclusion

The students were generally satisfied with the clarity, organization, and perceived fairness of the OSCE; however, concerns were raised about the relevance of stations to clinical practice and the adequacy of feedback. Despite positive perceptions of skill development, particularly in diagnostic and technical proficiency, mixed responses on critical thinking and identified challenges, such as time pressure, examiner training, suggest areas for improvement. Addressing these issues through targeted enhancements, including refining station relevance, incorporating clinical tasks, improving feedback mechanisms, and managing time constraints, can optimize the overall educational experience and ensure robust assessment of clinical competencies in dental education.

### Electronic supplementary material

Below is the link to the electronic supplementary material.

Supplementary Material 1

Supplementary Material 2

Supplementary Material 3

## Data Availability

The data included in the present study are available upon request from the corresponding author.
