# Supplementary material for: Analysis of satisfaction levels and perceptions of clinical competency: a mixed method study on objective structured clinical examinations in undergraduate dental students
Source: BMC Med Educ. 2024 Jun 17;24:673. doi: 10.1186/s12909-024-05639-0 (PMC11184762; doi:10.1186/s12909-024-05639-0)
Supplement: Supplementary file 2 — Supplementary Material 2 [file 12909_2024_5639_MOESM2_ESM.pdf]

<u>Consent form for participation in research study</u>

You are invited to participate in a research study titled "*Investigating the Effectiveness of Objective Structured Clinical Examinations (OSCEs) as an Assessment Method in Undergraduate Dental students: A mixed Methods study*." The purpose of this study is to assess student satisfaction levels and perceptions of clinical competency development through the implementation of OSCEs in dental education.

Your participation in this study is voluntary, and you have the right to withdraw at any time without any consequences. Your responses will be kept confidential and will only be used for research purposes. Please read the following consent statement carefully. By proceeding with the questionnaire, you indicate your voluntary agreement to participate in this research study.

Consent Statement:

I have read and understood the description of the research study provided above. I voluntarily agree to participate and provide my responses to the questionnaire. I understand that:

My participation in this study is voluntary, and I have the right to withdraw at any time without any consequences.

The information provided in the questionnaire will be kept confidential, and my responses will only be used for research purposes. The research findings may be reported in aggregate form, ensuring that individual participants cannot be identified. I have the right to skip any questions I do not wish to answer, and it will not affect my participation in the study. If I have any questions or concerns regarding the research study, I can contact the principal investigator whose contact information is provided below.

Principal Investigator:

[Prof.Dr.Naseer Ahmed and team]

[drnaseerahmed@altamash.pk]

Please select one of the following options:

- ○ I voluntarily agree to participate in this research study.
- ○ I do not wish to participate in this research study.

Participant Signature: ________________________

Date: _______________________

Questionnaire

Quantitative study data tool

Study title: Investigating the Effectiveness of Objective Structured Clinical Examinations (OSCEs) as an Assessment Method in Undergraduate Dental students: A mixed Methods study

Section 1: Demographic Information

Gender: Male / Female

Age: __________ years

Academic Year: __________

Previous OSCE Experience (if any): Yes / No

Section 2: Student Satisfaction

Please rate your level of satisfaction with the following aspects of the OSCE assessment method using a 5-point Likert scale (1 = Strongly Disagree, 2 = Disagree, 3 = Neutral, 4 = Agree, 5 = Strongly Agree):

2.1. The clarity of instructions provided before each OSCE station.

1 = Strongly Disagree, 2 = Disagree, 3 = Neutral, 4 = Agree, 5 = Strongly Agree

2.2. The organization and flow of the OSCE stations.

1 = Strongly Disagree, 2 = Disagree, 3 = Neutral, 4 = Agree, 5 = Strongly Agree

2.3. The fairness of the assessment process.

1 = Strongly Disagree, 2 = Disagree, 3 = Neutral, 4 = Agree, 5 = Strongly Agree

2.4. The adequacy of time provided for each OSCE station.

1 = Strongly Disagree, 2 = Disagree, 3 = Neutral, 4 = Agree, 5 = Strongly Agree

2.5. The relevance of the OSCE stations to the clinical practice.

1 = Strongly Disagree, 2 = Disagree, 3 = Neutral, 4 = Agree, 5 = Strongly Agree

2.6. The helpfulness of feedback received after the OSCE.

1 = Strongly Disagree, 2 = Disagree, 3 = Neutral, 4 = Agree, 5 = Strongly Agree

2.7. The overall effectiveness of OSCEs in assessing clinical competencies.

1 = Strongly Disagree, 2 = Disagree, 3 = Neutral, 4 = Agree, 5 = Strongly Agree

Section 3: Perceptions of Clinical Competency Development

Please rate your perception of the extent to which OSCEs have contributed to the development of the following clinical competencies using a 5-point Likert scale (1 = Not at all, 2 = Slightly, 3 = Moderately, 4 = Very much, 5 = Extremely):

3.1 Diagnostic skills.

1 = Not at all, 2 = Slightly, 3 = Moderately, 4 = Very much, 5 = Extremely

3.2 Treatment planning abilities.

1 = Not at all, 2 = Slightly, 3 = Moderately, 4 = Very much, 5 = Extremely

3.3 Technical proficiency in performing dental procedures.

1 = Not at all, 2 = Slightly, 3 = Moderately, 4 = Very much, 5 = Extremely

3.4 Communication skills with patients.

1 = Not at all, 2 = Slightly, 3 = Moderately, 4 = Very much, 5 = Extremely

3.5 Professionalism and ethical conduct.

1 = Not at all, 2 = Slightly, 3 = Moderately, 4 = Very much, 5 = Extremely

3.6 Critical thinking and problem-solving skills.

1 = Not at all, 2 = Slightly, 3 = Moderately, 4 = Very much, 5 = Extremely

3.7 Time management during patient care.

1 = Not at all, 2 = Slightly, 3 = Moderately, 4 = Very much, 5 = Extremely

3.8 Teamwork and collaboration with other healthcare professionals.

1 = Not at all, 2 = Slightly, 3 = Moderately, 4 = Very much, 5 = Extremely
