# Supplementary material for: Analysis of satisfaction levels and perceptions of clinical competency: a mixed method study on objective structured clinical examinations in undergraduate dental students
Source: BMC Med Educ. 2024 Jun 17;24:673. doi: 10.1186/s12909-024-05639-0 (PMC11184762; doi:10.1186/s12909-024-05639-0)
Supplement: Supplementary file 3 — Supplementary Material 3 [file 12909_2024_5639_MOESM3_ESM.pdf]

Qualitative study data tool

Group interaction will be based on the following list of topic questions pertaining to effectiveness of Objective Structured Clinical Examinations (OSCEs) in dental students.

1. Focused group discussion topics and questions:

**1. Introduction and Background:**
(P) Can you share your experiences with OSCEs as an assessment method in your undergraduate dental education?<br>(P) How many OSCEs have you participated in throughout your dental program?
**2. Perceived Benefits of OSCEs:**
(P) In your opinion, what are the advantages or benefits of using OSCEs as an assessment method in undergraduate dental education?<br>(P) How do you think OSCEs have contributed to your development of clinical competencies?
**3. Challenges and Difficulties:**
(P) What challenges did you encounter during the OSCEs in your undergraduate dental education? How did you overcome them?<br>(P) Were there any specific aspects of the OSCEs that you found particularly challenging? Why?
**4. Perception of Effectiveness:**
(P) How do you perceive the effectiveness of OSCEs in assessing your clinical competencies compared to other assessment methods?<br>(P) Do you think OSCEs adequately reflect real-life clinical scenarios? Why or why not?
**5. Integration into Curriculum:**
(P) How well do you think OSCEs are integrated into your dental curriculum? Are they well-aligned with your coursework and clinical experiences?<br>(P) Are there any specific suggestions you have for better integrating OSCEs into the dental curriculum?
**6. Feedback and Evaluation Process:**
(P) What are your thoughts on the feedback provided after the OSCEs? Did you find it helpful in your learning and development?<br>(P) How do you perceive the fairness and consistency of the evaluation process used in the OSCEs?

7. Quantitative Assessment vs. OSCEs:

(P) In your opinion, how do OSCEs compare to other forms of quantitative assessment (e.g., written exams) in evaluating your clinical competencies?
(P) Do you believe OSCEs provide a more accurate representation of your clinical skills? Why or why not?

8. Integration into Future Practice:

(P) How confident do you feel that the skills assessed through OSCEs will be applicable to your future dental practice?
(P) In what ways do you think OSCEs have prepared you for real-world clinical situations?

9. Overall Reflections:

(P) What are your overall thoughts and reflections on the use of OSCEs as an assessment method in undergraduate dental education?
(P) Do you have any additional comments or suggestions regarding the implementation or effectiveness of OSCEs?

P=prompts

2. In this study, the following transcription conventions will be used to convert the recorded audio or video data into written text.

**[[ ]]**	Overlap
**.**	Short pause
**M**	Moderator
**…**	Longer pause
**Ss**	Students
**S1**	Individual student
______________________	Talk inaudible on tape
**!**	Show surprise
**_**	Interrupted speech
**( )**	Moderator's insertion
**[ ]**	Elaboration of speech

3. **Dummy table for frequency of expressions on assessment in student focus group interviews**

Common student expressions on OSCE assessments	Frequency of occurrence in terms of percentages (N=45)
